# The Role of Oxytocin in the Dog–Owner Relationship

**DOI:** 10.3390/ani9100792

**Published:** 2019-10-12

**Authors:** Sarah Marshall-Pescini, Franka S. Schaebs, Alina Gaugg, Anne Meinert, Tobias Deschner, Friederike Range

**Affiliations:** 1 Domestication Lab, Konrad Lorenz Institute of Ethology, University of Veterinary Medicine, Savoyenstraße 1a, A-1160 Vienna, Austria; Alina.Gaugg@vetmeduni.ac.at (A.G.); Friederike.Range@vetmeduni.ac.at (F.R.); 2 Clever Dog Lab, Messerli Research Institute, University of Veterinary Medicine Vienna, Medical University Vienna, University of Vienna, 1210 Vienna, Austria; anne.meinert1990@gmail.com; 3 Max Planck Institute for Evolutionary Anthropology, Department of Primatology, Deutscher Platz 6, 04103 Leipzig, Germany; deschner@eva.mpg.de

**Keywords:** oxytocin, dog, human-animal relationship, social bond, attachment

## Abstract

**Simple Summary:**

A number of studies have shown that when dogs and humans interact with each other in a positive way (for example cuddling) both partners exhibit a surge in oxytocin, a hormone which has been linked to positive emotional states. It is not clear however, if this increase in oxytocin occurs between any dog and human or whether this is more specific to the dog–owner bond. In this study we measured oxytocin levels in dogs and humans before and after they interacted with their closely bonded partner (dog–owner dyads) and with a partner they were familiar with but with whom they did not have a close bond. Based on previous literature we predicted that dogs and owners would show an increase in oxytocin after a positive social interaction, and that this increase would be higher when the dog and owner were interacting with each other than when the interaction occurred with a partner that was just ‘familiar’. In fact, overall we did not find an increase in either, dogs’ or humans’ oxytocin level, although there was a lot of variability in the response. We discuss various reasons why our results are not in line with other studies.

**Abstract:**

Oxytocin (OT) is involved in multiple social bonds, from attachment between parents and offspring to “friendships”. Dogs are an interesting species in which to investigate the link between the oxytocinergic system and social bonds since they establish preferential bonds with their own species but also with humans. Studies have shown that the oxytocinergic system may be involved in the regulation of such inter-specific relationships, with both dogs and their owners showing an increase in OT levels following socio-positive interactions. However, no direct comparison has been made in dogs’ OT reactivity following a social interaction with the owner vs. a familiar (but not bonded) person, so it is unclear whether relationship type mediates OT release during socio-positive interactions or whether the interaction per se is sufficient. Here we investigated OT reactivity in both dogs and owners, following a socio-positive interaction with each other or a familiar partner. Results showed neither the familiarity with the partner, nor the type of interaction affected OT reactivity (as measured in urine) in either dogs or owners. Given the recent mixed results on the role of oxytocin in dog-human interactions, we suggest there is a need for greater standardization of methodologies, an assessment of overall results taking into account ‘publication bias’ issues, and further studies investigating the role of relationship quality and interaction type on OT release.

## 1. Introduction

Oxytocin (OT), a nine amino-acid neuropeptide, has remained highly conserved among vertebrate taxa throughout evolution [1]. In addition to its important role in sexual behavior, parturition and nursing [2], OT influences the amygdala, a central component of the neuro-circuitry regulating social cognition and fear [3]. In humans, OT has been shown to be a key mediator of emotional and social behaviors such as attachment [4], social recognition [5], and in-group/out-group dynamics [6,7]. A similar involvement of OT in the mother–infant as well as sexual-partner bonding has been shown also in a number of non-human species [1,8,9]. More recently, OT has also been shown to be elevated when interacting with preferred social partners such as “friends” [10]. For example, a study conducted on wild chimpanzees found that urinary OT levels increased after grooming bouts, but this effect was mediated by the quality of the affiliative bond between partners in that regardless of the genetic relatedness or sexual interest between partner, the closer the affiliative bond, the higher the OT levels following the grooming session [11]. 

Dogs are a particularly interesting species in which to investigate the potential links between the oxytocinergic system and social bonds, since aside from establishing differential bonds with conspecifics [12] there is growing evidence that this species can form close relationships to specific human partners, which have been functionally equated to the mother–infant attachment bond [13,14,15,16,17]. Indeed, a number of studies reported that dogs display an owner-specific secure base effect similar to that described in human children, for example showing an increase in exploration and play behavior [13,18] as well as exhibiting higher success rates in a problem solving task when in the presence of their owner compared to a stranger [19]. Furthermore, Gácsi et al. [20] found that dogs reacted with more confidence and showed less signs of stress in a threatening situation, when their owner was present suggesting that the owner functions as a “safe haven” for dogs. 

Also from the humans’ perspective, there is ample evidence that owners form a close emotional bond to their dogs. Stoeckl and colleagues [21] conducted functional magnetic resonance imaging studies showing that the presentation of both human and dog family members’ faces activated the same brain region. Interestingly, these brain areas are known to interact with the OT system. Furthermore, a more recent study [22] also confirmed the activation of a common network of brain regions involved in emotion, reward, affiliation, and social cognition both when mothers viewed images of their child and their dog. 

These findings raise the question of the mechanisms that enable the formation of such strong relationships between dogs and their human caregivers and several studies suggest that the oxytocinergic system may play a crucial role [15,23,24,25,26,27]. In two studies Nagasawa and colleagues [24,27] measured OT levels in urine of both dogs and owners following social interactions between them. Dogs were clustered in two groups depending on the length of time they spent looking at their owners (LG—long gaze, or SG—short gaze), and it was observed that following the social interaction with their dog, owners of LG dogs showed an increase in urinary OT concentration compared to pre-treatment levels. This in turn reinforced owners’ affiliative interaction with the dogs (i.e., duration of stroking and talking), which also resulted in an increase in OT levels in dogs compared to pre-treatment levels. Authors concluded that results support the existence of an OT-mediated positive feedback loop facilitating dog–owner bonding. From the humans’ perspective, several other studies have shown that plasma OT levels of owners increase following positive social interactions with their dogs (stroking, playing, and talking) [15,23,28], further supporting the potential involvement of OT in the maintenance of the dog–owner bond.

However, more recently a number of authors have criticized the oversimplified view of OT’s role in social relationships in general [29] and in the dog-human relationship in particular [17]. Indeed, findings from the dogs’ perspective are inconsistent, with dogs’ endogenous OT levels being shown to increase following social interactions with their owner in some studies [15,27,30] but not in others [26,31]. Furthermore, in some studies dogs’ OT levels increased also after being stroked by a familiar person rather than the owner [22,32] calling into question the link between oxytocin and bonding. Furthermore, it is not clear which aspects of the dog-human social interaction elicit increased OT levels in dogs. In line with the importance of tactile contact in eliciting OT release in humans and mammals in general [33], stroking has been shown to elicit OT in some studies [22,23,25], but mutual gazing appears to be sufficient in others [24,27]. Similarly, from the human side, it is not clear whether it is just the sensory stimulation of stroking a dog, which triggers OT release [33] or whether it is in fact the social interaction, and more specifically the social interaction with ones’ own dog, which plays the central role. The latter possibility would be in line with the hypothesis that OT is mediated by relationship quality at least in some species [11]. Indeed, perhaps surprisingly, to date, no study has actually compared whether OT levels in humans and dogs differ following a positive social interaction with their bonded companion in comparison to a familiar dog/person.

Hence in the current study, we aimed to examine a number of the hypotheses suggested so far in the literature: we tested the ‘relationship quality hypothesis’ by comparing the increase in endogenous OT levels (measured in urine) in both dogs and owners following a socio-positive interaction involving stroking, talking, and mutual eye contact (cuddle condition) with either their bonded partner or a familiar non-bonded individual. Furthermore, to evaluate whether dogs’ OT levels would in fact increase more as a result of a social interaction than in its absence (‘social interaction hypothesis’), the cuddle condition with the owner/ familiar person was compared to an equivalent condition in which the owner/familiar person was cuddling a furry toy dog (fake dog condition) and not giving attention to the “real” dog. This condition also allowed us to investigate if the act of stroking itself, regardless of the dogs’ identity (own vs. familiar dog) or whether it is real or fake (fake vs. own/familiar dog), leads to changes in OT levels in humans. Additionally, to test for the possibility that in dogs, increase in OT levels are simply linked to the aspect of tactile stimulation and not to the social interaction with the person, we also conducted a condition, where dogs were “stroked” by an artificial hand held by the owner without engaging in a social interaction (i.e., talking and gazing) (mechanical cuddle condition). For each condition, urinary OT levels in both dogs and humans were measured before and after the respective interaction and the change between pre- and post-treatment levels was evaluated.

If the ‘relationship quality hypothesis’ is correct, i.e., in both, dogs and humans, changes in OT levels during an affiliative interaction are mediated by strength of the social bond, we expect a greater increase in the OT levels from pre- to post-treatment in both dogs and humans after interacting with their bonded companion (the owner/own dog) compared to after interaction with the familiar non-bonded dog/person.

If the ‘social interaction hypothesis’ is correct, i.e., affiliative interactions involving stroking, gazing and talking lead to increased OT levels in both dogs and humans, then in both dogs and humans, we should observe a greater rise in OT levels from before to after treatment in conditions in which a human (owner/familiar person) interacts in a friendly way by cuddling the dog (own/familiar dog) compared to non-social control conditions with the owner/familiar person being present but ignoring the dog (fake dog condition).

Finally, if instead tactile stimulation per se affects OT levels and social interaction is not a crucial element, we would predict a) no significant differences in the change of dogs’ OT levels when the dog is being cuddled by their owner directly whilst socially engaged with it (owner cuddle condition) vs. when the owner uses an artificial hand and no social interaction occurs (mechanical cuddle condition) and b) differences in dogs’ OT levels should be more pronounced in the cuddle conditions (owner and familiar) compared to the non-social control conditions without interaction/stimulation (owner/familiar with fake dog).

## 2. Material and Methods

### 2.1. Study Design

All subjects gave their informed consent for inclusion before they participated in the study. The study was conducted in accordance with the Declaration of Helsinki, and the protocol was approved by the Ethics Committee of the University of Veterinary Medicine, Vienna (Approval number: ETK-16/05/2016 and extension ETK-12/05/2018) for dogs, and from the Medical University of Vienna for humans (Approval number: 1565/2016 and extension 1573/2018).

In the present study, we tested 20 dog–owner dyads (dogs: 10 males, 10 females, mean age 6.1 years, age range: 2–11 years see Table 1 for details of breed). Since previous studies showed that OT in female dog owners tended to increase more than in male participants [28,34], we included only female dog owners, who were healthy and neither menstruating, pregnant nor lactating. A precondition for dogs was that they were familiar and at ease spending time at the Clever Dog Lab, and were all equally familiar with the experimenter (AM or AG), who acted as the ‘familiar’ person in the experiment. We used a within subject design and conditions were conducted in a randomized and counterbalanced order across subjects. Between conditions there was a gap of at least three days for both dogs and humans.

### 2.2. Experimental Setup

All experiments were conducted using an indoor room at the Clever Dog Lab (CDL) of the University of Veterinary Medicine Vienna. Urine samples of the dogs were collect outside of the lab on the campus of the university; one before (pre-treatment—D1) and one after each session (post-treatment—D2). The bathroom in the CDL was used to collect human pre-treatment urine samples (H1) as soon as they entered the CDL as well as post-treatment samples (H2) just before the owner left the lab for collecting the dog post-treatment samples. All urine samples were stored in a −20 °C freezer and sent, on dry-ice, to the Max Planck Institute for Evolutionary Anthropology, Department of Primatology (MPI EVA) in Leipzig, Germany for analysis.

**Figure 1:**
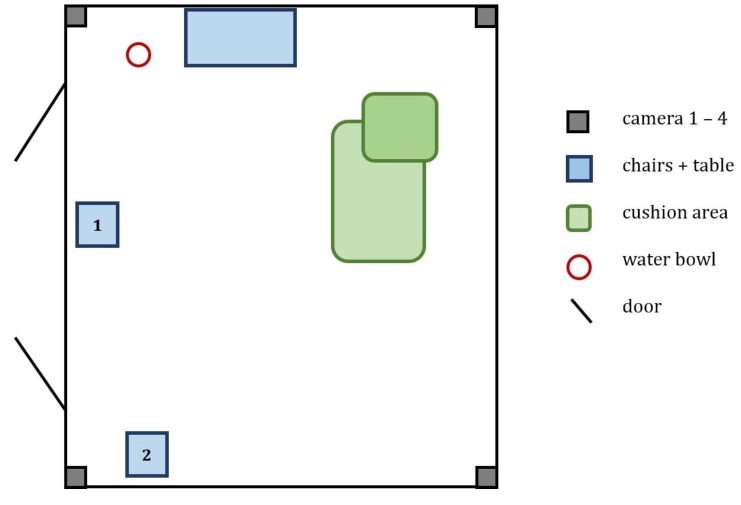
Setup of the indoor testing room.

The indoor room was equipped with two chairs, a table, blankets and cushions on the ground and a water bowl for the dog. Chair 1 was occupied by the owner during the initial 5 min of each session, whereas after treatment the owner sat at the desk and worked on her computer. During the whole experiment, the experimenter was present in the room, sitting quietly on chair 2 and not interacting either with the dog or the owner. In the familiar person condition the positions were reversed. There was an alarm activated by the experimenter to indicate the end of each time phase. All test sessions where video-recorded, using 4 video cameras placed in the corners of the room to ensure full coverage of the room (Figure 1).

The familiar person (AM or AG) was known to the dog, but did not live in the same household, did not carry out any activity with it and contact between them was restricted to sporadic interactions no more than approximately 10 min per week when meeting in the offices of the university prior to testing.

The same restrictions applied to the familiar dogs used in the tests. The owners (of the test dogs) knew the familiar dogs, but did not live with them and had only had sporadic contact with them when meeting in the university offices prior to testing. Thus, the dog was a familiar but non-bonded individual to the owner. The familiar dog was of the same sex as the owner’s dog.

#### 2.2.1. Pre-Testing Instructions to Owner and Pre-Sample Collection

**Figure 2:**
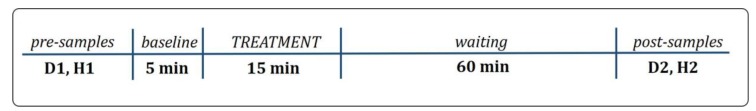
Chronology of the experimental phases.

Preceding the experiment, owners were asked to not engage in any social interactions (cuddling, playing) with their dogs and insure that they and their dogs did not engage in any exercise or feeding activities for at least 60–90 min before the experiment started, since this could have an influence on the pre-treatment OT level. Dog and owners were however together during the time preceding the test. Owners and their dogs were first met in the outdoor area where the D1 urine sample was collected. Afterwards, the experimenter guided the dog and the owner to the CDL indoor area and collected the H1 urine sample. Following the sample treatment (see Section 2.2.3. Urine Sampling Collection and Treatment) and storing of the samples, the session started (Figure 2).

#### 2.2.2. Treatment Procedure

The test consisted of three phases: 1) a 5 min “baseline”, 2) a 15 min “treatment”, followed by 3) a 1 h “waiting” phase (Figure 2). In all conditions, the baseline phase consisted of a period in which the dog could freely explore the room, whilst the owner/familiar person and experimenter quietly sat in the chairs, not interacting with the dog. Following this, either the owner or the familiar person moved to the cushion area and the treatment phase started, which in most cases (see below) involved an interaction time between the dog and the owner/familiar person. The exact treatment depended on the condition (Table 2).

##### Owner Cuddle

Whilst the experimenter remained sitting on the chair reading a book or working on the computer and ignoring both, owner and dog, the owner moved to sit comfortably on the cushions. She was allowed to call the dog over to the cushion area and start an interaction in a positive, calm way: Gentle stroking or massaging, talking to the dog, looking into the dog’s eyes etc. The owner was instructed to have a cuddle session as natural for them as possible, allowing the dog to relax. If the dog moved away, the owner was allowed to gently call it back a maximum of three times. The owner remained sitting in the cushion area for 15 min and spent as much of that time as possible gently stroking the dog, but never forcing the dog to remain there against its will. For this condition urine samples were collected from both owner and dog.

##### Familiar Cuddle

Analogous to the Owner cuddle condition, but in this case the owner remained seated and the experimenter (i.e., the familiar person) got up and sat on the cushions and initiated the cuddle session. The type of gentle interaction was as similar as possible to that of the owner. Hence prior to testing the experimenter asked the owner where the dog likes to be stroked, and how. For this condition urine samples were collected only from the dog.

##### Owner Fake Dog

During the 5-min baseline, the owner held the furry toy dog in her hands/lap whilst sitting on the chair and the dog was allowed to sniff and explore the furry toy. If the dog attempted to hold, grab, play with it, the owner gently placed a hand between the dog and the toy, to stop/prevent this occurring. After the baseline period, whilst the experimenter remained sitting in the chair, the owner sat comfortably in the cushion area but in this condition, she stroked a furry toy dog and did not interact with her dog in any way—so no talking, touching or looking at the dog. Instead, the owner was asked to interact with the furry toy in a way as similar as possible to the way of interacting with their dog (including gently talking to it and looking it in the eyes). If the dog tried to initiate an interaction, the owner just continued stroking the furry toy. Urine samples were collected from both owner and dog.

##### Familiar Fake Dog

Analogous to Owner fake dog condition, but carried out with the familiar person instead of the owner, whilst the owner sat quietly on the chair. Urine samples were collected only from dog.

##### Owner Cuddle Other Dog

Analogous to Owner cuddle condition, but carried out with a familiar dog instead of the owner’s dog. The owner’s dog was completely absent during this condition. Here, urine samples were collected only from the owner.

##### Owner Mechanical Cuddle

An artificial hand was used to “stroke” the dog providing tactile stimulation while the social aspect of the interaction was absent. In fact, when stroking the dog with the artificial hand, the owner was asked to do so while facing away from the dog and without talking to it. Furthermore, to further avoid potential eye-contact, the owner wore dark sun-glasses. Urine samples were collected from both owner and dog.

In all conditions, the interaction time between the owner/familiar person and dog/fake dog was kept as constant as possible (i.e., unless the dog was uncomfortable and moved away, the interaction lasted 15 min). In the final waiting phase, the owner/familiar person returned to the chair after treatment and read a book or worked on the computer and no longer paid attention to the dog. After this period the H2 and D2 urine samples were collected.

#### 2.2.3. Urine Sample Collection and Treatment

Dog urine samples were collected using a closable plastic cup attached to a stick (polypropylene, Carl Roth, CEN 7.1). Human participants were asked to use same kind of plastic cup.

Prior to sampling, Eppendorf vials were prepared with 100 µL phosphoric acid, then 1 mL of urine was transferred into each vial, manually shaken for several seconds and labelled with a unique code, ID of the subject, date and time. Aliquots were created, if the experimenter managed to collect extensive amounts of urine. The vials were stored in a −20 °C freezer.

#### 2.2.4. Attachment Questionnaire

The owners were asked to answer an attachment questionnaire before starting the first test session. We used the Lexington Attachment to Pets Scale (LAPS), since it is one of the most validated questionnaires regarding the owners’ attachment to their pets [35]. The questionnaire comprises 23-items; examples of questions include: “My pet and I have a very close relationship” and “I feel that my pet is a part of my family”. A 5-point scale (1 = strongly agree to 5 = strongly disagree, with 3 = neutral) was adopted. Several questions are reverse scaled, so reverse scoring was adopted prior to computing the final score. The correlation between the calculated attachment score and the increase of the OT levels in the owner cuddle condition for both owners and dogs helps to illuminate the possible influence of relationship quality.

#### 2.2.5. Behavior Coding

The duration of social interaction between the human and the dog (including the fake dog) was coded during the treatment phase of the test sessions. The duration of social interaction was a composite category including the duration of stroking, gazing and talking to the dog. Furthermore, we coded the dogs’ stress-related behaviors i.e., the summed frequency of lips-licking and yawning. The video-coding of the dog and human behavior was carried out using the Solomon Coder (copyright: András Péter, www.solomoncoder.com). Interobserver reliability was conducted on 20% of the data (coded both by AG and AM) and was found to be acceptable (Intraclass Correlation ICC on duration of: stroking 0.986; talking to the dog 0.712; gazing at the dog 0.73 ICC on frequency of: yawning 0.759, lips licking 0.823).

#### 2.2.6. Laboratory Analyses

Analysis of the urine samples for OT levels was conducted in the Endocrinology laboratory at the MPI EVA in Leipzig, Germany. The analyzer was blind regarding the conditions the samples belonged to. The methodology for the analysis of urinary OT levels in both dogs and humans was validated beforehand [36,37]. Urine samples were extracted following [11]. In brief, after thawing the samples at 4 °C, they were gently vortexed for 10 s and centrifuged for 1 min at 1500 rpm. For all dog samples, 250 µL urine were mixed with 750 µL of 0.1% trifluoracetic acid (TFA) and for humans 1 mL of urine was mixed with 1 mL 0.1% TFA. Following conditioning of the SPE cartridges (Chromabond HR-X, 1 mL, 30 mg) with 1 mL methanol (HPLC grade, 100%) and 1 mL water (HPLC grade), urine samples were loaded on the cartridge and washed with 5 mL 10% (vol/vol) acetonitrile (ACN) containing 1% TFA in water. Samples were eluted with 1 mL 80% (vol/vol) ACN. Extracts were dried down at 50 °C using a gentle stream of compressed air, reconstituted in 300 µL ethanol (100%), incubated for 1 h at 4 °C after gently vortexing for 10 s and dried down again at 50 °C. Finally, all dried extracts were reconstituted in 250 µL of OT assay buffer (which was supplied with the commercial assay kit, Enzo Life Sciences Assay designs (Enzo Life Sciences, Basler Strasse 57a 79540 Lörrach Germany), Cat. No. 901-153A-0001), leading to dog urine samples being measured at 1:1 and human urine samples at 4:1 concentration. Reconstituted samples were gently vortexed for 10 s, centrifuged for 1 min at 10,000 rpm and measured in duplicates following the instructions given by the assay supplier (Assay designs, Cat. No 901-153A-0001). To compensate for the variation in the volume and concentration of the voided urine, we measured creatinine concentrations in each urine sample and express all oxytocin values as pg mg^−1^ creatinine. In total 10% of the measurements were below the linear range of the assay, however, these were still kept in the study as we found that higher levels of concentration (e.g., re-measuring samples at 4:1 instead of 1:1) leads to an underestimation of OT levels (for further details see [36]. We excluded samples of one human (condition owner fake dog) as the pre test sample for this condition was too high to be measurable with the assay system. Inter-assay coefficients of variation were 15.2% for a high and 8.2% for a low-quality control (N = 22). Intra-assay coefficient of variation was 10.9% (N = 29; following guidelines by Salimetrics). Please see [36] for a detailed analytical validation of OT measurements in urine samples of dogs and [37] for humans.

#### 2.2.7. Statistical Analyses

Based on our hypotheses, specific differences were predicted to occur in dog’s and owner’s oxytocin levels depending on condition. In all conditions attempts were made to keep the duration of the interaction treatment constant. However, because this could not be guaranteed (some dogs moved away), we included the duration of interaction as a test variable where relevant for both the dog and owner models. Furthermore, oxytocin is known to be interrelated with the HPA-axis [38,39,40] and although we pre-selected dogs that were known to be comfortable with social interactions, we nevertheless measured their stress-related behaviors and also included these as a control factor in the dog models.

##### Models on OT Variance in Dogs

To assess the effect of human–dog interactions on dog urinary oxytocin levels, we fitted two general linear mixed models (LMM, [41]) with urinary oxytocin levels (log-transformed) as response variable.

Since for two (Familiar fake dog and Owner fake dog) of the five conditions there was no interaction between the human and the dog (the owner and a familiar person were interacting with the fake dog and not the real one), we fitted one model including the complete data set, but lacking duration of interaction as test variable (dog model I) and a second model with comprising only the three remaining conditions (Owner cuddle, Familiar cuddle, and Owner mechanical cuddle) including also duration of interaction as test variable (dog model II).

For the first model (dog model I), we included condition (Owner cuddle, Familiar cuddle, Owner mechanical cuddle, Owner fake dog, Familiar fake dog) and treatment (pre- and post-urine collection) as test variables [42] with fixed effects. To investigate whether changes in OT levels following treatment were different across conditions, we also included a two-way interaction between the respective main effects. Sex of the dog, time of day and frequency of stress-related behaviors were included as control variables [42] with fixed effects and subject ID as well as partner ID included as random effects. Random slopes were included for condition and pre/post within subject ID and partner ID and for frequency of stress-related behaviors within partner ID.

For the second model (dog model II), on a subset of the dataset including only the conditions Owner cuddle, Familiar cuddle, and Owner mechanical cuddle, we included duration of interaction, condition and treatment (pre- and post-urine collection) as test variables [42] with fixed effects. To test for a differential effect of the conditions in relation to the duration of interactions on changes in OT levels, we included a three-way interaction between the respective main effects. We included sex, time of day and frequency of stress-related behaviors as control variables [42] with fixed effects and subject ID as well as partner ID included as random effects. We included random slopes for condition, pre/post and duration of social interaction within subject ID and partner ID.

For all dog and human models, we z-transformed the following fixed effects to a mean of zero and a standard deviation of one [43]: Time of day, duration of interaction and frequencies of stress-related behaviors. To allow for their inclusion as random slopes, duration of interaction, and treatment (pre- and post-urine collection) were centered to a mean of zero. By visual inspection of a qqplot and residuals plotted against fitted values, we checked for the assumptions of normally distributed and homogeneous residuals. Model stability was assessed by excluding levels of the random effects one by one and comparing the estimates of the model derived for these fixed effects with the ones obtained for the full dataset. We checked for collinearity using the Variance Inflation Factor [44] using the function vif of the R package car [45] by fitting a standard linear model excluding random effects and interactions. Collinearity revealed to not be an issue for any of our models (maximum VIF human model: 3.63; dog model I: 1.23; dog model II: 1.32). We fitted all models using the function lmer of the lme4 R package [46] using Maximum Likelihood. Likelihood ratio tests were used to establish the significance of the full model when compared to the null model [47,48]; all control factors and the random effects were kept in the respective null models and likelihood ratio tests were used to assess significance of the individual effects [49]. In case the full-null model comparison revealed to be significant but interactions included in the full model revealed to be non-significant, such interactions were removed to enable easier interpretation of the main effects [43]. All models were fitted using R ([50] R core team, version 3.6.0. (R Core Team, Vienna, Austria, https://www.R-project.org/).

##### Models on OT Variance in Owners

To assess the effect of human–dog interactions on human urinary oxytocin levels we fitted a general linear mixed model (LMM, [41]) with urinary oxytocin levels (log-transformed) as response variable. We included condition (Owner cuddle own dog, Owner cuddle other dog, Owner mechanical cuddle own dog, Owner cuddle fake dog) and treatment (pre- and post-urine collection) as test variables [42] with fixed effects. To assess whether changes in urinary oxytocin levels were dependent on the condition, we also included a two-way interaction between the respective main effects. Duration of interaction was included as control variable [42] with fixed effects and subject and partner ID included as random effects. To keep the type I error rate at the nominal level of 5% [49,51] we included random slopes of all fixed effects within subject and partner ID. We had to exclude one human urine sample in the fake dog owner condition due to unusually high OT levels. Therefore, final N for human samples is 20, except for this one condition (fake dog condition) where it is 19.

## 3. Results 

Overall, dog (N = 20) oxytocin levels pre-treatment showed a mean of 65.61 pg/mg creatinine and ranged from 18.42 to 218.18 pg/mg creatinine. Post-treatment, mean oxytocin values were 68.19 pg/mg creatinine, ranging from 5.27 to 266.77 pg/mg creatinine. 

Whilst in the owner cuddle condition 8 out of 20 dogs showed an increase in OT levels from pre to post treatment of over 10% (less than 10% was considered as a normal variation) (mean = 55.3%, range = 10.2–107.4%), only 5 dogs had higher post treatment OT levels in the Familiar person cuddle condition (mean = 72.9%; range = 36.4–119.5%). In the Owner mechanical hand condition 7 dogs showed higher OT levels post treatment (mean = 41.7%; range = 12.3–110.7%), 4 dogs showed an increase in OT levels following the Familiar fake dog condition (mean = 51.6%; range = 22.3–106.1%) and 8 dogs showed an increase in the Owner fake dog condition (mean = 59.9%; range = 10.5–198.7%).

Results based on the complete dataset (dog model 1) showed no interaction or main effect of condition, treatment or frequency of stress related behaviors on the dogs’ oxytocin levels (full model not significantly different to the null model; χ^2^ = 6.41, df = 9, *p* = 0.698). Similarly, results of the dog model 2, considering only variables in which an interaction between a human and the dog occurred (Owner cuddle, Familiar cuddle, and Owner mechanical cuddle), showed no effect of treatment, condition or the duration of the interaction full model not significantly different to the null model; χ^2^ = 8.45, df = 11, *p* = 0.673).

Overall, owner oxytocin levels pre-treatment showed a mean of 52.37 pg/mg creatinine and ranged from 5.78 to 871.52 pg/mg creatinine. Post-treatment, the mean oxytocin value was 58.76 pg/mg creatinine, ranging from 2.99 to 1037.12 pg/mg creatinine. In comparison to dogs, increases in post-treatment OT levels of humans were generally more pronounced (Owner cuddle condition (N = 13)—mean: 174.67%; range: 10.2–580.29%; Familiar cuddle condition (N = 10)—mean: 321.3%; range: 17.3–1632.3%; Mechanical hand owner condition (N = 14)—mean: 89.09%; range: 22.8–314.1%; Fake dog owner condition (N = 12)—mean: 83.3%; range: 15.05–271.4%). Results for the owner model showed no interaction or main effect of treatment or condition (full model not significantly different from the null model; χ^2^ = 8.57, df = 7, *p* = 0.285).

**Figure 3:**
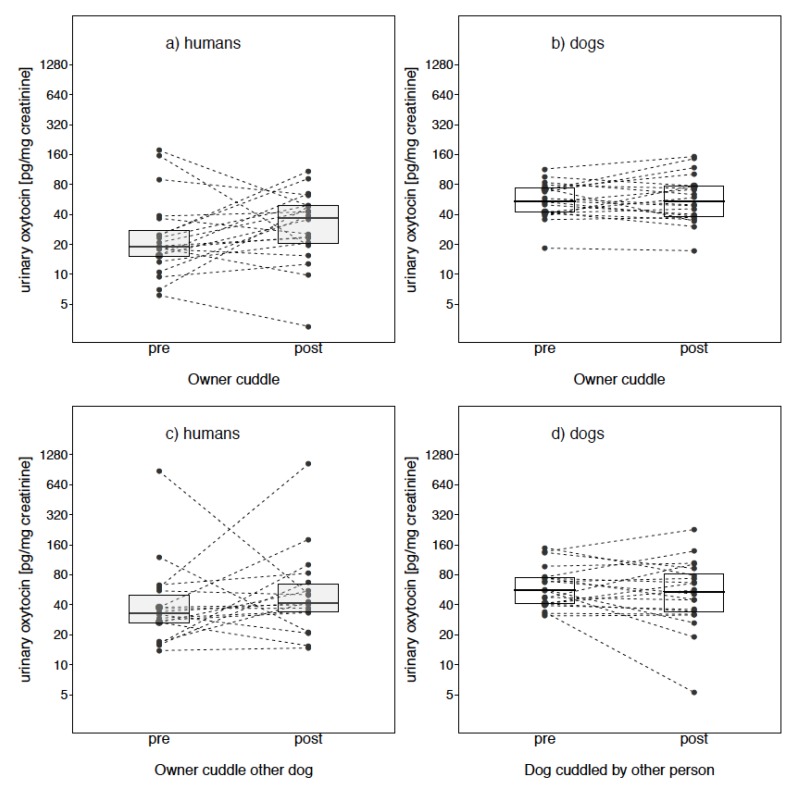
Oxytocin levels for owners (**a**,**c**) and dogs (**b**,**d**), pre- and post-treatment in the Owner cuddle condition, in which owner’s are cuddling their own dog (**a**,**b**), and in the Owner cuddle other dog (**c**) and dog cuddled by a familiar person (**b**) condition.

**Figure 4:**
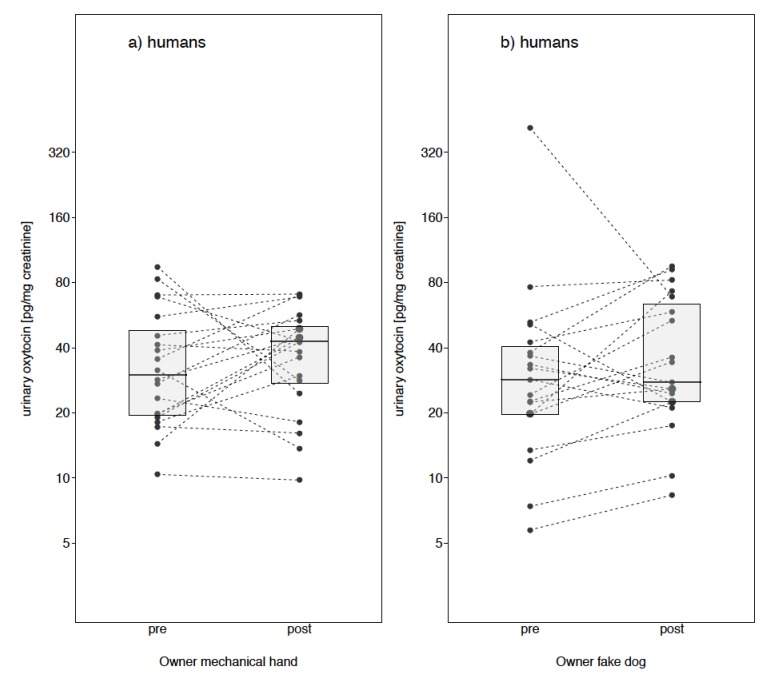
Oxytocin levels pre- and post- treatment for owners in the Owner fake dog condition (**a**) and Owner mechanical hand condition (**b**).

Taken together results suggest that overall, oxytocin levels in dogs and humans were unaffected by our treatment conditions. However, visual inspection reveals that the owner’s oxytocin levels were much more variable (as compared to dog OT levels) in response to interacting with their own dog, stroking it with an artificial hand, a familiar dog and even a furry toy dog (see Figure 3 and Figure 4).

Results of the attachment questionnaire showed a mean of 4.02 (range 3–4.52). No correlation (Spearmen’s Rho) emerged between the questionnaire score and either the change in oxytocin levels from pre- to post- owner cuddle for the owner (R = 0.05), or the dogs (R = 0.05).

## 4. Discussion

Taken together results of the current study are puzzling. In contrast to most other studies, we were unable to show that a socio-positive interaction with either a bonded or familiar partner significantly increased peripheral oxytocin levels in either *dogs*, or their *owners*.

Dog OT levels, as measured in blood, were found to increase following an interaction with a person (owner or familiar) [15,23,25,30,32] and in all such studies, social interactions involved either talking, eye contact, and stroking or just stroking for between 5 and a maximum of 30 min. Indeed, in the one study that compared these eliciting stimuli, physical contact involving stroking was shown to best trigger a rise in oxytocin [25]. Fewer studies have been conducted using measures of oxytocin in urine and saliva. Nevertheless, Mitsui et al. [22] found that dog’s OT levels measured in urine (and blood) increased after a 15 min stroking session with a familiar person and in another study, in at least a subset of dogs (those showing longer gazing times to the owner), it was found that mutual gaze and touching from the owner increased oxytocin compared to pre-treatment levels [27]. Furthermore, OT measured in dog saliva, also showed an increase following social interaction (gently petting, talking and eye contact) with a familiar person [32]. Similarly previous studies found an increase in human’s OT both when measured in urine [24,27] and blood [15,23,28], following a socio-positive interaction. Hence, from a behavioral perspective, the social interactions between owner/familiar person and dog in the current study appear sufficiently comparable to most previous procedures to justify a prediction that such an interaction would elicit an increase in both the dogs’ and the humans’ oxytocin levels. 

A number of potential explanations should be considered for the current lack of an effect, some relate specifically to the dogs, whereas others may be applicable to both species. First, the breed of the dogs may have an influence on oxytocin reactivity. Although at present there are no studies showing a different oxytocin reactivity to social interactions across breeds, there is some evidence that breeds may respond differently to OT administration (Huskies vs. Border collies; [52]), and may differ in allele frequency for certain OXTR polymorphisms (Beagles Border Collies, German Shepherds, Golden Retrievers, Groenendaels, Hungarian Vizslas, Labrador Retrievers, Malinois, Siberian Huskies and Tervueren; [53]). However, our current study population was composed of predominantly mixed breed dogs and a variety of the pure-breed dogs (see Table 1), thus the breed composition of our population is unlikely to account for the lack of a treatment effect on oxytocin levels. Moreover, our sample size is similar to most previous studies measuring endogenous oxytocin after a social interaction with a person. 

Another possibility is that although all dogs were familiar with both the testing location and the experimenter acting as the ‘familiar’ individual, we inadvertently selected dogs that although tolerating the contact, were not overly relaxed or comfortable with it. This could explain the greater variability in oxytocin levels observed when the familiar person was carrying out the cuddle session and the decrease in oxytocin levels from pre to post treatment in seven of the 20 animals tested in this condition (Figure 3d). Moreover, the study was based on the assumption that dogs have a stronger relationship with their owner than with a familiar person they see only occasionally, and that such a bond has a positive valence. However, owner relationships styles with their dog can vary from ‘warm’ to more ‘controlling’ with clear impacts on the dogs’ behavioral reactions in stressful situations [54]. Indeed, in a questionnaire-based study, a correlation emerged between mean oxytocin levels (measured in blood samples of 10 male Labrador retrievers following a 3 min social interaction with the owner) and questionnaire items related to the intensity of the owner–dog relationship (as measured by the Monash Dog Owner Relationship Scale—although see [31] for no such association using the same tools) [55]. Hence, inadvertently, we might have selected dog–owner dyads with a weak, or not particularly ‘warm’ social bond. Although we cannot entirely exclude this possibility, we think this is an unlikely explanation since results from the ‘Lexington pet-attachment questionnaire’ showed that the reported attachment was in the upper range of the scale for all owners, and there was no correlation between the magnitude of change in oxytocin levels for either dogs or owners in the owner cuddle condition and the results of the questionnaire. 

One other, more methodological reason for the discrepancy with previous studies is related to the analyses carried out in the current study to evaluate dogs’ and humans’ oxytocin levels. Indeed the analyses used for urine samples in previous studies were based on radioimmunoassays (RIA—[56,57,58]) whereas in the current study we used an enzyme immunoassay (EIA). Different assay systems might be more or less susceptible to matrix effects (substances in the sample matrix that might interfere with the assay system). These matrix effects can lead to falsely high or low OT values, and these effects might only be present at certain sample concentrations. It has been argued that EIA’s are more susceptible to matrix effects than RIA’s. Usually, extraction procedures aim to eliminate these substances from the samples, and assay system validation procedures check for these effects by conducting a test for parallelism. In addition, depending on the assay system, the antibody that is used can have additional immunoreactivities with structurally similar substances that also bind to the antibody (so called cross-reactivities). These cross-reactivities can lead to different results in OT measurements that are not caused by OT itself but depend on the amount of cross-reactive substances present relative to OT. Finally, different assay systems can differ in their sensitivity, i.e., the lowest amount of the hormone of interest they are able to detect. Taken together, these different assay system characteristics (which are usually reported by the assay manufacturer and/or identified by analytical validation procedures) can lead to considerable differences in OT measurements of the same sample when measured with different assay systems. Therefore, care should be exercised when comparing and/or drawing conclusions across studies that measured samples with different assay systems, both in terms of the absolute values and the overall patterns of results found across conditions. 

Interestingly, a recent study also using the Enzo EIA to assess the potential increase in dogs’ OT as measured in urine following either a walk or a social interaction session with their owner, also found no effect [31], potentially suggesting that this methodology suffers from greater limitation compared to the RIAs. Nevertheless, we do not think the different methodologies used can fully account for the lack of OT increase in the dogs. Firstly, there is at least one study using RIA, which also did not show the predicted increase in dogs’ OT levels following a positive interaction with the owner [26], suggesting that the effect of socio-positive interactions on OT may not necessarily be as consistent as initially thought. Second, because an in depth analytical validation process was carried out for the oxytocin extraction and analyses of both dog and human samples used in the current study and on the basis of a number of parameters (e.g., test for parallelism, extraction efficiency, assay accuracy, immunogram to assess immunoreactivity, and repeatability), validity of the assay was shown to be equally reliable for dog and human samples [36,37]). Nevertheless, even though the assay system was validated, some general issues relating to repeatability and storage of samples emerge. More specifically, when dog and human urine samples were stored and re-analyzed at different time periods (after 1 months, 3 months, 5.5 months) [36,37]) we found that oxytocin values extracted from the same samples were not consistent over time, and they did not show a uniform pattern of change across and within individuals that could be statistically accounted for. Considering samples from this study were analyzed at different time periods after the collection (mean = 133.4 days, SD 55.4), this may have affected the overall pattern of results. It is therefore possible that some of these effects added noise to the dataset thereby masking smaller effects potentially linked to the testing condition.

A second methodological issue, which is also an on-going debate in both the animal and human literature, is to what extent peripheral measures of oxytocin meaningfully reflect changes in central levels [59]. The underlying assumption is that peripheral oxytocin measures reflect central levels due to coordinated release in both the brain and periphery following a socio-positive interaction [9,60]. Indeed, a number of studies in human and non-human animals have found support for coordinated release in both central and peripheral areas (rats: [61,62]; female prairie voles: [63]; humans: [64,65]). However, other studies have reported no significant association between releases in the two areas (guinea-pigs: [66]; rhesus monkeys: [67]; humans: [68,69]). In the most recent review of the topic, [70] carried out an in depth meta-analyses and found that peripheral oxytocin (as measured in blood) correlated with central levels following oxytocin administration but *not* at baseline. Such results would suggest that whereas peripheral oxytocin may be useful when detecting a particularly high increase in oxytocin (e.g., in the case of administration), it may be less reliable in detecting more subtle changes following less impacting treatments. Furthermore, correlation between oxytocin levels in urine and blood have also received mixed results, with some studies showing positive correlations (e.g., dogs: [22]) but others reporting none (humans: [71,72]).

A third, more specific issue, is how adequate urinary measures of oxytocin are for dogs. In fact, measures of oxytocin in urine may be particularly difficult to assess because dogs’ urination normally does not involve a complete emptying of the bladder. Although we explicitly asked owners to not carry out activities known to affect oxytocin levels prior to arrival at the lab, it is still possible that without the complete emptying of the bladder prior to treatment, any oxytocin increase would be diluted in the urine already present in the bladder, and potentially affected by prior activities, making the detection of an existing effect more unreliable. Although both issues outlined above need to be considered, it is also important to note that two previous studies have shown an increase in dogs’ urinary oxytocin following social interactions with a person, suggesting that looking at peripheral OT is a valid method in dogs. So, taken together, while it seems that urinary oxytocin can be used as an adequate monitoring method, it may be more difficult to detect less striking changes in oxytocin levels, than for example measures of OT taken from blood samples, and that this method may be more difficult to apply to dogs than other species.

A similar issue may have caused some problems with the oxytocin measures in the human population. Indeed in other studies [24,27], participants were asked to arrive at the lab 2 h prior to testing and asked to empty their bladders one hour prior to test and spend this time quietly resting. Although we gave complete instructions to owner regarding their behaviors prior to the test, it is still possible that in our study the pre-sample still contained the influence of other ‘uncontrolled for’ factors, thereby making the pre-post treatment changes harder to detect.

Current results suggest that the influence of socio-positive interactions with people on dogs’ oxytocin levels is not particularly strong and/or consistent. Indeed, considering the four published studies comparing urinary measures of oxytocin before and after social interactions with a person, only one found a consistent increase (n = 9) [22], a second study found that only a subset of dogs looking longer at the owner (n = 8 of the 30 tested) showed an oxytocin increase [27], and the third and fourth study found no effect at all (n = 16, [26]; n = 30, [31]). The first three studies used the same analyses methods (RIA); yet the consistency of the effect across studies was low. Powell et al. [31] and the current study rather used EIA, and in both cases no increase was detected. 

The overall picture for increase in OT levels in humans after a positive interaction with a dog is also far from clear. When considering OT measured in human urine sample an increase was found after a positive social interaction with their own dog in only a subset of individuals in one study (in 13 of 55 individuals tested; [24]. Increase in OT in blood appears a bit more consistent with two studies reporting this effect in female subjects (8 out of 10 women, but none of the 10 men showed an increase in [28]; and overall in 10 females tested, no individual data provided [15]), and one reporting the effect in both men and women (N = 18, but no individual data provided [23]).

In light of these results and given the potential for “publication bias” where only studies showing significant effects are reported (see [73]), the role of OT in the mediation of the dog-human relationship might be currently overrated.

Furthermore, a lack of consistent findings across studies might be due to limited sample sizes in some studies. Conducting a power analysis before running a study may help to identify a sufficient sample size in advance. However, given the complexity of our study (compared to studies that have been conducted on that topic so far) and given the fact that most published studies do not report detailed enough data on, e.g., actual oxytocin levels and individual differences, we found that conducting an informative power analysis would not be possible. Post hoc power analysis, in turn, actually does not provide any more information than the p-value itself [74,75]. An alternative, however, is to investigate confidence intervals once the study has been carried out (CIs) [74]. The CIs in our study (see Supplementary Material Figures S1–S3) reveal the estimates to be of considerable uncertainty, suggesting that although current sample sizes are in line with previous studies, they may be too low to detect meaningful patterns and/or factors other than the ones we investigated here may have a considerable effect on OT levels.

Given the variance in the OT response in our dog (and although to a lesser extent also the human) population, it may be the case, that the standardized social interaction we required from owners in our test conditions in the lab, may have been an OT activating exchange for some dog–owner dyads but not others. Potentially a more naturalistic study, in which owners are asked to interact with their dogs “as they do normally” during quiet times together in their home environment and familiar persons then reproduce a similar interaction in the same setting may result in stronger effects relating to OT release.

Overall, given the relatively few studies currently published on the effect of human–dog interactions on oxytocin levels in both species, the mixed results so far obtained and the substantial differences in the laboratory analyses used, we need to be careful in drawing any conclusions in regard to the involvement of OT release in the human–dog relationship, and its hypothesized role in human- animal assisted programs [76]. We encourage a more concerted effort to standardize procedures and replication as well as the publication of results also where treatment effects were not found. This is especially important in a newly evolving research area that includes the measurement of OT levels from non-invasively collected matrices, such as urine samples, to avoid the erroneous impression of a well-established method in a still developing field.

## Figures and Tables

**Table 1:** Participants in the study (breed based on owner questionnaire information).

Dog	Sex	Castration	Age (Years/Months)	Breed	Owner
Ch	female	Yes	8/3	Australian Shepherd	JS
Cl	female	Yes	2/4	Mixed breed	DB
Gu	female	No	11/7	Border Collie	FR
Hy	female	Yes	8/3	Mixed breed-likely labrador mix	JE
Lo	female	No	3/9	Mixed breed-likely herding mix	DB
Ti	female	Yes	9/8	Mixed breed-husky mix	SM
Tu	female	Yes	2/7	Mixed breed	JE
Fr	male	Yes	2/8	Mixed breed-likely terrier mix	GC
Li	male	No	2/3	Australian Shepherd	JS
Ki	male	Yes	6/9	Mixed breed	RD
Ma	male	No	11/10	Golden Retriever	SM
Mo	male	Yes	3/6	Mixed breed-likely labrador mix	AM
Ol	male	Yes	10/5	Mixed breed-likely herding mix	KG
Sc	male	Yes	2	Mixed breed-likely herding mix	SK
Tia	female	No	5/2	Border Collie	AG
Pa	female	Yes	3/8	American Staffordshire Terrier	SJ
Me	male	Yes	8/6	Border Collie	MK
Che	female	No	4/3	Australian Shepherd	CG
Ja	male	Yes	8/5	Labradoodle	HLJ
Jac	male	Yes	6/10	Mixed breed—Greece shepherd mix	KR

**Table 2:** Overview of the treatment conditions and pre- and post- treatment samples collected for the dog and owner.

Category	Condition	Owner’s Dog	Owner	Dog Sample	Owner Sample
Social Conditions	Owner cuddle	Is being cuddled by their owner	Is cuddling their own dog	yes	yes
	Familiar cuddle	Is being cuddled by the familiar person	Is quietly sitting in the room	yes	no
	Owner cuddle other dog	Dog-subject is absent, another ‘stooge/familiar’ dog takes its place	Is cuddling a familiar dog	no	yes
Non-social Conditions	Owner fake dog	Is present but not given attention to	Is cuddling a fake dog	yes	yes
	Familiar fake dog	Is present but not given attention to	Is quietly sitting in the room	yes	no
	Owner mechanical cuddle	Is being stroked by the artificial hand held by the owner. No social interaction.	Is stroking (but no other social interaction) own dog using an artificial hand	yes	yes

## Supplementary Materials

The following are available online at https://www.mdpi.com/2076-2615/9/10/792/s1, Figure S1: Confidence Intervals for Model 1 testing for the effect of condition (Owner cuddle, Familiar cuddle, Owner mechanical cuddle, Owner fake dog, Familiar fake dog) and treatment (pre- and post-urine collection) and the interaction between them, on dogs’ oxytocin levels. Figure S2: Confidence Intervals for Model 2, on a subset of the dataset including only the conditions Owner cuddle, Familiar cuddle, and Owner mechanical cuddle. We tested the effect of the duration of interaction with the person, condition and treatment (pre- and post-urine collection) and the three-way interaction between them on dogs’ oxytocin levels. Figure S3: Confidence Intervals for Model 3, testing the effect of condition (Owner cuddle own dog, Owner cuddle other dog, Owner mechanical cuddle own dog, Owner cuddle fake dog) and treatment (pre- and post-urine collection) and their interaction on the owner’s oxytocin levels.
