# Supplementary figures and images for: The Role of Oxytocin in the Dog–Owner Relationship
Source: Animals (Basel). 2019 Oct 12;9(10):792. doi: 10.3390/ani9100792 (PMC6826447; doi:10.3390/ani9100792)

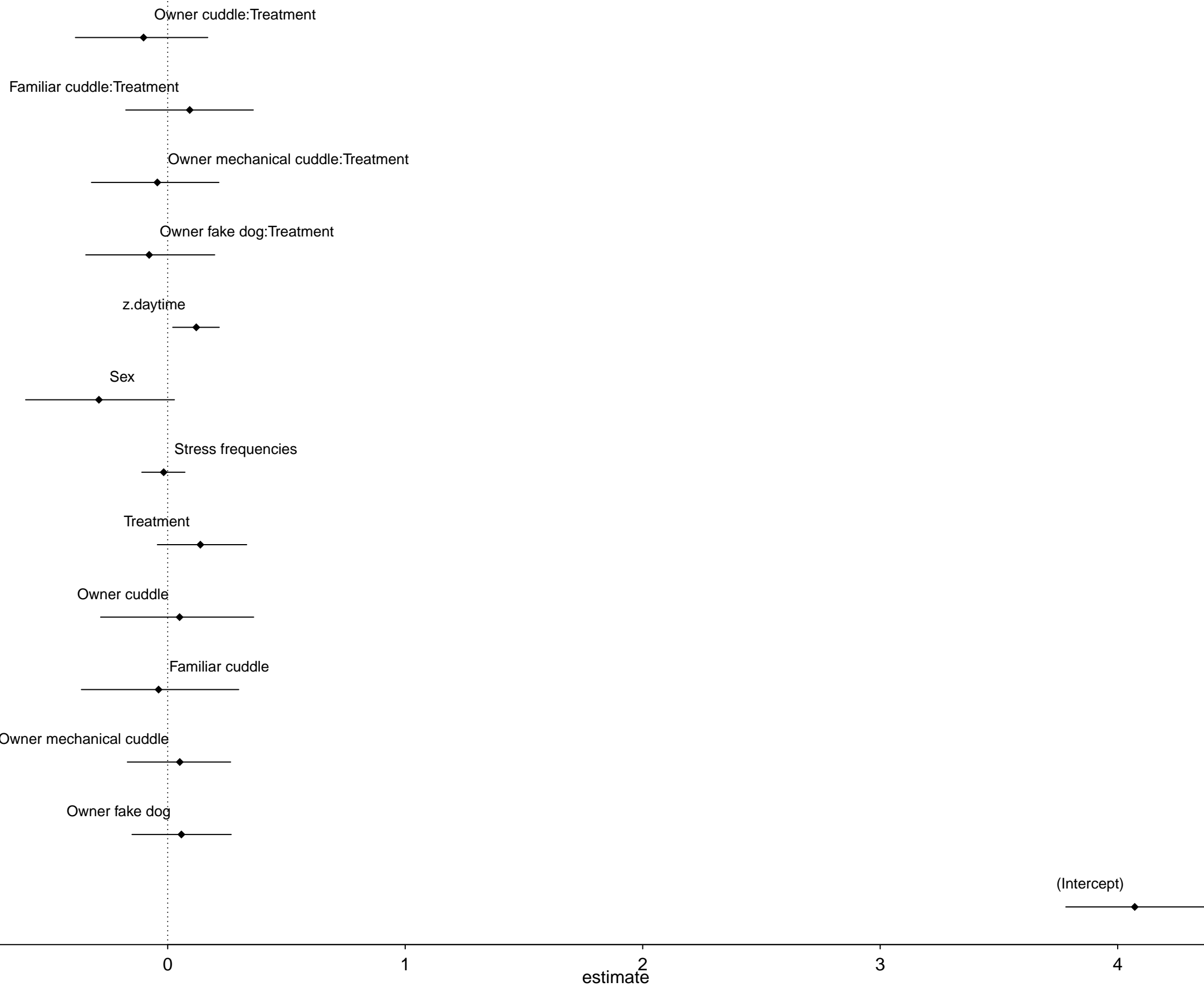

Supplement: Supplementary file 1 [file animals-09-00792-s001.zip › Supp Fig. 1 CI_dog model 1.pdf]

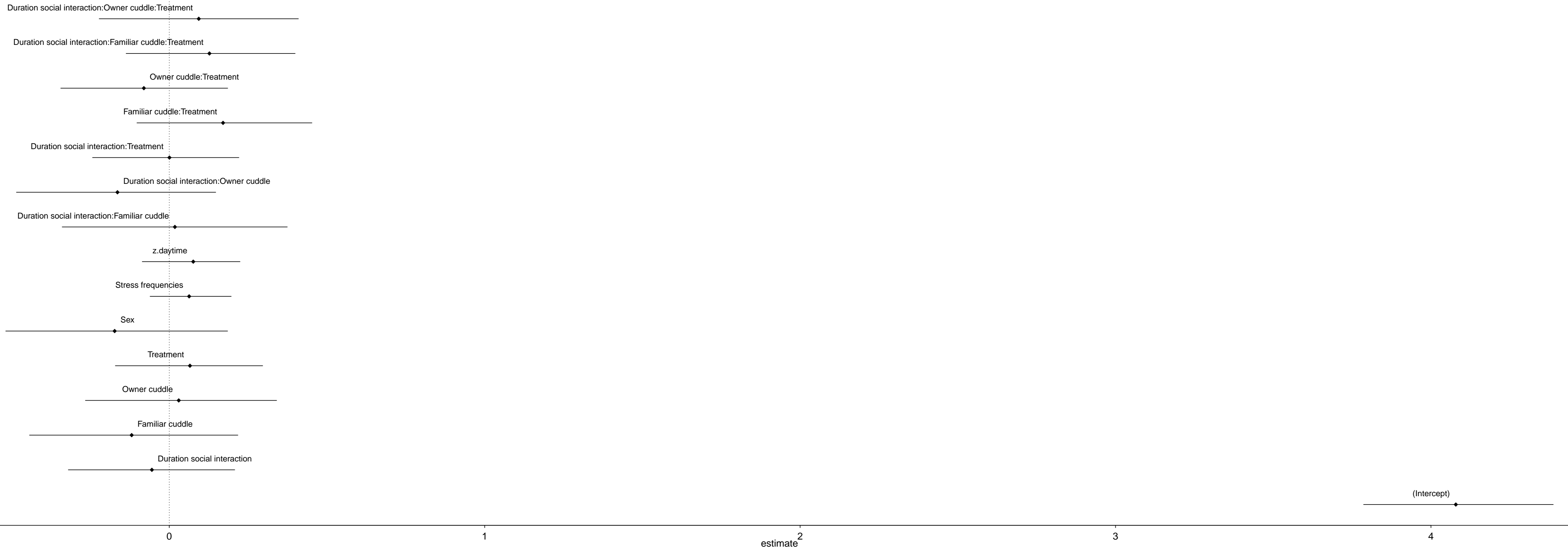

Supplement: Supplementary file 1 [file animals-09-00792-s001.zip › Supp Fig. 2 CI_dog model 2.pdf]

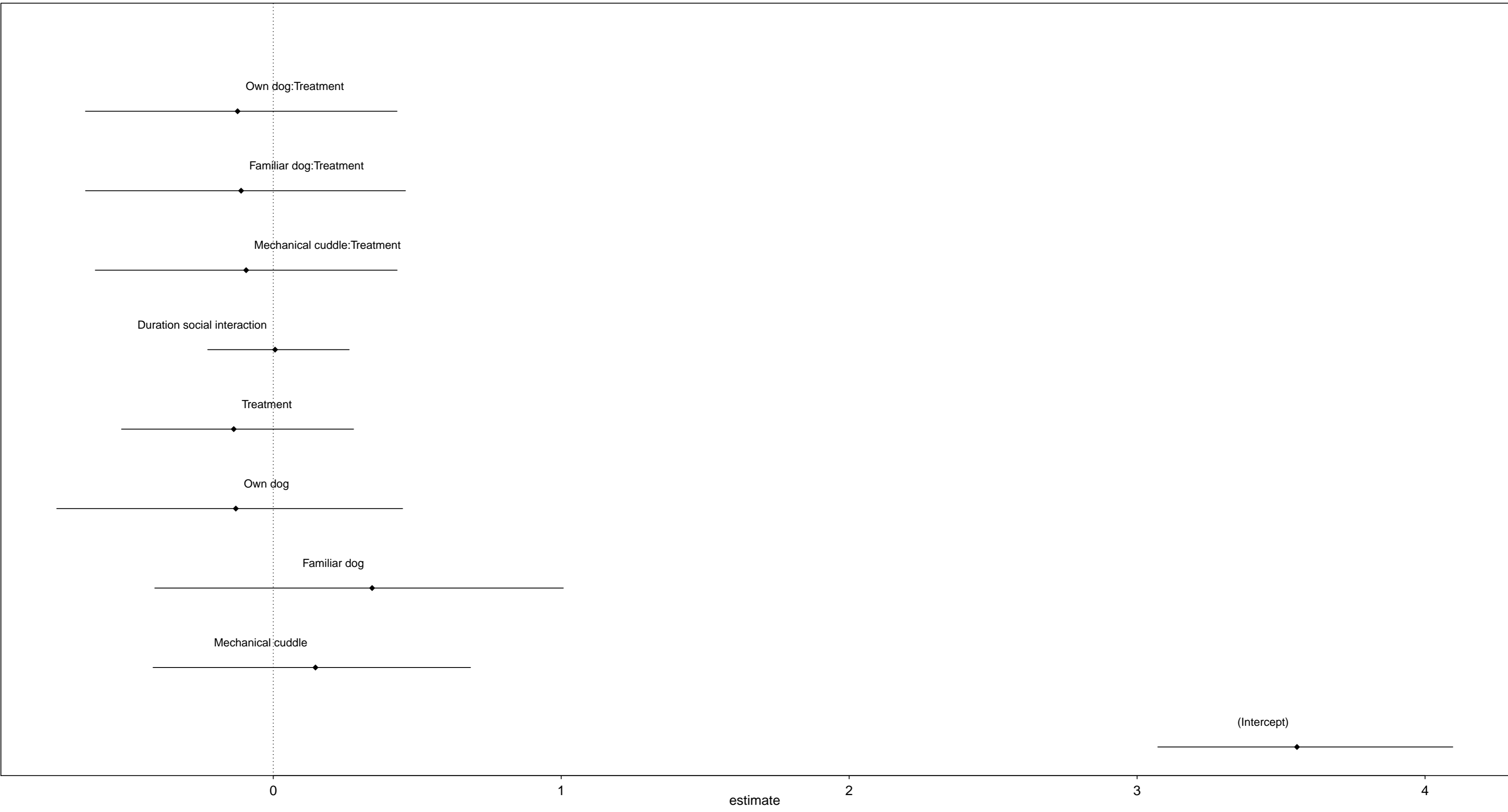

Supplement: Supplementary file 1 [file animals-09-00792-s001.zip › Supp Fig. 3 CI_human model.pdf]
